# eHealth cognitive rehabilitation for brain tumor patients: results of a randomized controlled trial

**DOI:** 10.1007/s11060-021-03828-1

**Published:** 2021-09-06

**Authors:** Sophie D. van der Linden, Geert-Jan M. Rutten, Linda Dirven, Martin J. B. Taphoorn, Djaina D. Satoer, Clemens M. F. Dirven, Margriet M. Sitskoorn, Karin Gehring

**Affiliations:** 1 Department of Neurosurgery, Elisabeth-TweeSteden Hospital, Tilburg, the Netherlands; 2 Department of Cognitive Neuropsychology, Tilburg University, Room S219, P.O. Box 90153, 5000 LE Tilburg, the Netherlands; 3 Department of Neurology, Leiden University Medical Center, Leiden, the Netherlands; 4 Department of Neurology, Haaglanden Medical Center, The Hague, the Netherlands; 5 Department of Neurosurgery, Erasmus Medical Center, Rotterdam, the Netherlands

**Keywords:** Cognitive rehabilitation, eHealth, Glioma, Meningioma, Neurosurgery, Randomized controlled trial

## Abstract

**Background:**

Evidence-based cognitive rehabilitation programs for brain tumor patients are not widely available, despite the high need. We aimed to evaluate the effects of a tablet-based cognitive rehabilitation program on cognitive performance, cognitive complaints, fatigue, and psychological distress in primary brain tumor patients following neurosurgery. Also, attrition, adherence and patient satisfaction with the program were evaluated.

**Methods:**

Adults with presumed low-grade glioma and meningioma were recruited before surgery. Three months thereafter, participants were allocated to the intervention group or waiting-list control group using minimization. The 10-week eHealth app *ReMind*, based on the effective face-to-face intervention, consisted of psychoeducation, strategy-training and attention retraining. Performance-based cognitive outcomes and patient-reported outcomes were assessed before surgery and 3, 6 and 12 months thereafter. Mean scores, percentages of cognitively impaired individuals and reliable change indices (RCIs) were compared between groups.

**Results:**

Sixty-two out of 183 eligible patients were randomized. Of the people who declined, 56% reported that participation would to be too burdensome. All participants found a tablet-app suitable for delivery of cognitive rehabilitation and 90% rated the program as “good” or “excellent”. Performance-based cognitive outcomes and patient-reported outcomes did not significantly differ in group means over time nor RCIs between the intervention (final *n* = 20) and control group (final *n* = 25).

**Conclusions:**

Recruitment at this early stage was difficult, resulting in limited statistical power. No significant effects were demonstrated, while adherence and satisfaction with the eHealth program were good. In clinical practice, *ReMind* may be helpful, if timing would be adapted to patients’ needs.

## Introduction

Cognitive deficits are common in adults with primary brain tumors [1–6]. Despite the fact that these cognitive deficits are often mild and diffuse in nature, they can lead to problems in social and professional functioning, which affect families and society [7–9]. In addition, brain tumor patients often face severe fatigue, distress and/or language problems [10–14], which may all contribute to lower quality of life. Patients with meningioma and patients with glioma with favorable prognosis [15, 16] in particular, live longer with a variety of symptoms, including cognitive deficits. Therefore, treatment of cognitive deficits has become increasingly important in the management of the disease [17].

The few studies that have been conducted on cognitive rehabilitation in adults with brain tumors demonstrated positive effects on cognitive outcomes in patients in different stages of the disease [18–23]. In a previous randomized controlled trial (RCT) of our group, patients with glioma who underwent cognitive rehabilitation performed significantly better on tests of memory and attention, and reported less cognitive complaints and mental fatigue afterwards [19, 20]. To increase the accessibility of this cognitive rehabilitation program [24] in a cost-efficient and patient-friendly way, we developed a tablet-based version of the program. An initial pilot study demonstrated that post-surgical cognitive rehabilitation via this eHealth intervention was feasible in adults with low-grade glioma and meningioma [25].

In this RCT, we investigated the effects of the tablet-based program in adults with low-grade glioma and meningioma, cognitive performance as primary outcome and self-reported cognitive functioning, fatigue, and psychological distress [26] as secondary outcomes. Enrollment, attrition, adherence, and patient satisfaction were evaluated as well.

## Methods

The study was conducted in accordance with the Declaration of Helsinki [27] and was approved by the local medical ethical review board (METC Brabant: NL51152.028.14). All participants provided written informed consent. Greater methodological details were described in a previously published study protocol [26] and feasibility study [25].

### Participants

Adult patients with presumed low-grade glioma or meningioma scheduled for resective surgery were screened for eligibility at the Elisabeth-TweeSteden Hospital Tilburg, Haaglanden Medical Center The Hague, and Erasmus Medical Center Rotterdam. People were not eligible if: they had multifocal disease or multiple brain tumors; had undergone brain tumor resection in the last year; received chemotherapy or radiotherapy in the last two years; had a history of progressive neurological disease/severe psychiatric disorder or substance abuse; had been diagnosed with an acute neurological/psychiatric disorder in the last two years; lacked a basic proficiency in Dutch; had an IQ below 85; had a KPS below 70; or had visual, language or motor impairment limiting the ability to complete neuropsychological assessment. Participants were also excluded after surgery if they suffered from surgery-related complications or if they were referred to conventional cognitive rehabilitation.

With the use of G*Power, an a priori power analysis for F-tests was conducted, to determine the minimum required sample size. The analysis indicated that, with alpha set at 0.05, power at 0.80 and an effect size of 0.37 (based on effect sizes of our previous RCT), group sizes of 50 were required (100 participants in total). With an expected attrition rate of 33 %, we aimed to include 150 participants before surgery and to evaluate data from 100 participants.

### Design, randomization and procedure

Patients in this multicenter prospective RCT were invited to participate prior to surgery. After patients’ approval, but before randomization, informal caregivers were also invited to participate and to support patients with the intervention. Participants underwent neuropsychological assessments before surgery (T0) and three months after surgery (T3). At T3, participants were assigned to the intervention group or to the waiting-list control group in a 1:1 ratio. The minimization method [28] was used to balance groups for age, tumor histology, baseline cognitive test performance, physical health status (ASA score) and participation in other psychosocial interventions [26]. Access to an online minimization program was provided by the Netherlands Cancer Institute Amsterdam [29]. With the use of this software, the allocation *sequence* remained concealed from the researcher. However, the researcher was not blinded and assigned participants to the intervention. Research assistants who carried out the neuropsychological assessments were blinded. Neuropsychological follow-up assessments were conducted immediately after the intervention (6 months post-surgery; T6) and one year post-surgery (T12). Participants in the waiting-list control group were offered the opportunity to follow the cognitive rehabilitation program, with guidance from the researcher, after the last study assessment.

### Intervention

The tablet-based cognitive rehabilitation program *ReMind* includes psychoeducation, strategy training and an attention retraining game. The psychoeducational information and strategy training are spread over six modules, namely (1) Cognitive functions, (2) Influences, (3) Compensation, (4) Attention, (5) Planning & Control, and (6) Memory. In each module, information about cognitive functions is given. Subsequently, compensatory strategies are provided, together with fill-in exercises to practice with the strategies in daily life. For example, patients learn to minimize distraction and to optimally use external devices for support. The modules include several user-friendly technical features, for example the possibility of using videos/audio clips in addition to written information, to look up frequently-used terms, and to send e-mails to the caregiver or professional if the patient gets stuck. The retraining includes game-like hierarchically graded exercises aimed at training different forms of attention (i.e., Sustained, Selective, Alternating and Divided attention). It includes visual and auditory exercises, wherein both verbal and numeric stimuli are presented. The *ReMind*-app works on iOS systems of iPad devices (Apple Inc) and is not yet publicly available.

The advice (presented in a leaflet) to patients was to spend three hours a week (spread over the week) on the program to complete the program in ten weeks. Telephone assistance was provided by the researcher every 2 weeks. During this telephone contact, participants could share their questions, difficulties, and experiences, and they were also encouraged to continue working on the program.

### Outcome measures

The primary outcome of this RCT was change in performance-based outcomes. Secondary outcomes were changes in patient-reported outcomes (PROs) and tertiary outcomes were enrolment and attrition, adherence and patient satisfaction.

#### Enrollment and attrition

The number of patients invited to participate was recorded, as were the numbers of patients who agreed or declined, and the reasons for decline and dropout.

#### Adherence and patient satisfaction

The number of completed modules in the strategy training and the number of completed exercises in the retraining, each expressed in percentages, were used to measure adherence to the program. If a strategy training module was not fully completed, module sections were counted. Adherence was considered sufficient if patients completed ≥ 80 % of both the strategy training and the retraining. Experiences with the program were evaluated with a study-specific evaluation questionnaire [25].

#### Performance-based cognitive outcomes

Cognitive functioning was measured with the computerized neuropsychological test battery Central Nervous System Vital Signs (CNS VS, LCC, Morrisville, North Carolina) [30]. CNS VS assesses the following domains: verbal memory, visual memory, processing speed, psychomotor speed, reaction time, complex attention and cognitive flexibility. Additionally, working memory was assessed with the Digit Span Test of the Wechsler Adult Intelligence Scale (WAIS-III), and verbal fluency was measured with a Letter Fluency test [31]. Patient scores were converted to *Z*-scores (correcting for age, sex and/or education) using Dutch norms [31–33]. Impaired cognitive functioning was defined as *Z*-scores ≤− 1.5.

#### Patient-reported outcomes (PROs)

The Cognitive Failures Questionnaire (CFQ) was used to measure self-reported cognitive failures. Additionally, two index scores of the Behaviour Rating Inventory of Executive Function (BRIEF-A) were evaluated, namely Behavioral regulation and Metacognition. Two subscales of the Multidimensional Fatigue Inventory (MFI-20) were also analyzed to evaluate the level of physical fatigue and mental fatigue. Symptoms of anxiety and depression were examined using the Hospital Anxiety and Depression Scale (HADS). Scores were converted to *Z*-scores based on published norms [34–37] and, *Z-*scores ≤− 1.5 were considered as low.

### Statistical analysis

Statistical analyses were conducted using SPSS version 24 (IBM Inc, Armonk, New York), with alpha set at 0.05.

Regarding enrollment and attrition, analysis of non-response bias was conducted, to explore possible differences between people who declined participation and people who provided informed consent at T0. Subsequently, pre-intervention (T3) sociodemographic, clinical and neuropsychological characteristics were compared between the intervention and control group using independent sample *t*-tests, Chi-square tests or Fisher’s exact tests.

To study the performance-based cognitive outcomes and patient-reported outcomes, we used repeated measures ANOVAs to assess effects of time and group on the outcome variables. In addition, chi-square tests were conducted to evaluate frequencies of participants with impairment (*Z*-scores ≤− 1.5) at each time-point. (i.e., T3, T6, T12).

Reliable change indices (RCIs) were calculated for the cognitive test scores and PROs, by comparing change in individual scores to observed changes in the study’s control group, while taking into account practice effects, natural recovery and measurement errors [38]. Reliable improvement was defined as RCI values above + 1.645 and decline below − 1.645 (based on an alpha of 0.10, corresponding to a 90% confidence interval). RCIs were calculated over the first time-interval (T3–T6) and over the second interval (T3–T12). Numbers of participants who reliably improved/declined on one or more outcomes were compared between groups for test scores and PROs using Chi-square tests.

## Results

### Enrollment and attrition

**Fig. 1:**
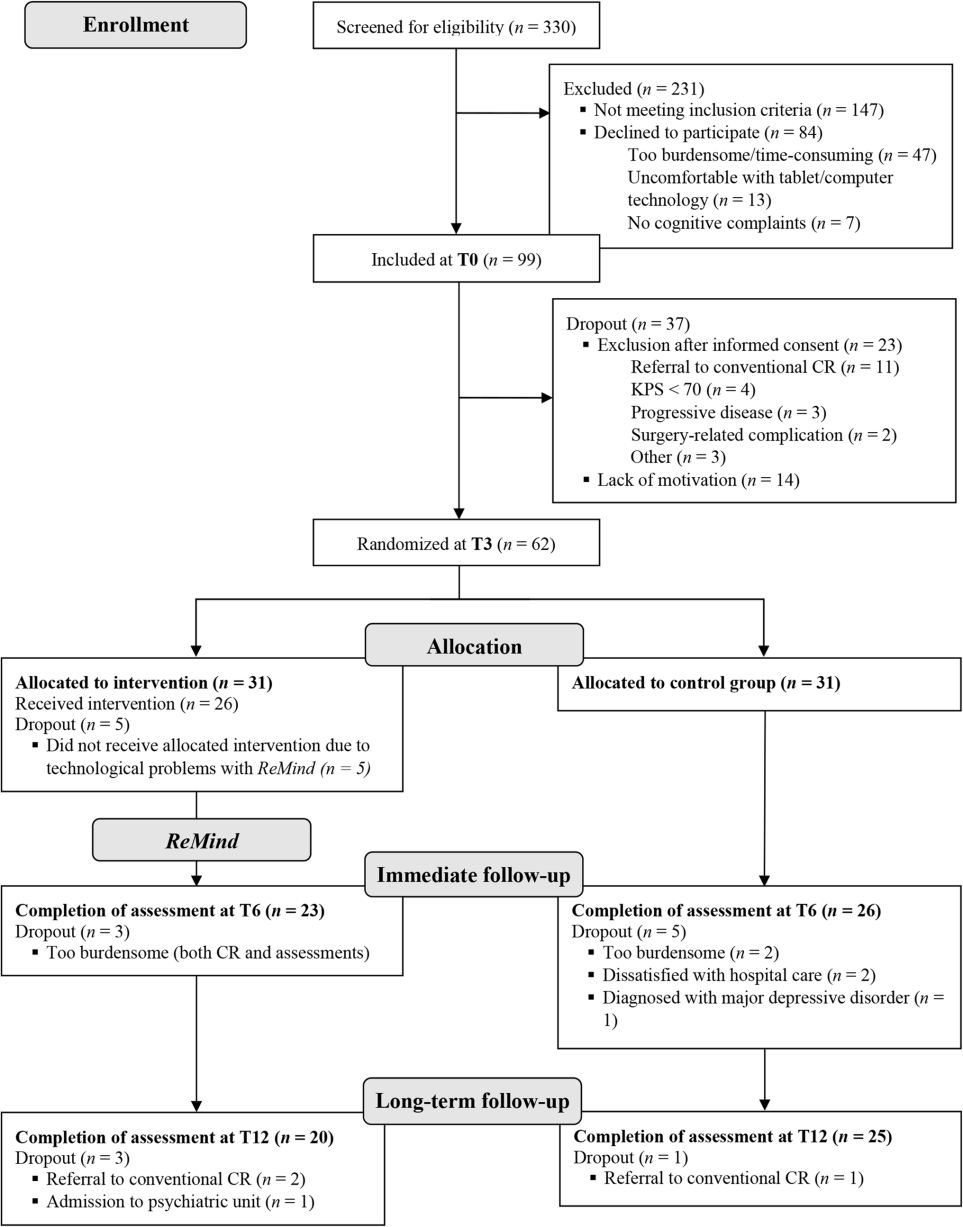
Flow of participants through the trial. *CR* cognitive rehabilitation; *KPS* karnofsky performance status. Neuropsychological assessments took place one day before surgery (T0), and 3 (T3), 6 (T6) and 12 (T12) months thereafter

Figure 1 presents the flow of participants throughout the trial. Prior to surgery, 183 out of 330 patients were eligible based on the inclusion/exclusion criteria and were invited to participate, of whom 99 provided informed consent and 84 declined participation. The most important reason for decline was that patients anticipated that it would be too burdensome and/or too time-consuming (*n* = 47; 56 %). Non-response analysis with available data of 75/84 decliners showed that study participants were significantly younger (M_diff_=-8.09, *p* = .03), were more often highly educated (50% vs. 20%, *p* < .05) and were more often diagnosed with a LGG (32% vs. 11%, *p* < .05) compared to non-responders. No significant differences were observed in sex, tumor lateralization or proportions of patients with cognitive impairment.

From T0 to T3, before randomization, 37 participants dropped out of the study. Most important reasons were lack of motivation (*n* = 14; including 8 patients who wanted to devote full attention to work resumption) and referral to conventional cognitive rehabilitation (*n* = 11). Three months after surgery, 62 participants were randomized to the intervention or control group. In total, 17 participants dropped out of the study between T3 and T12 for various reasons (see Fig. 1). As a result, data of 49 participants were included at T6 and of 45 participants at T12.

### Patient characteristics

Participants in the intervention group were significantly younger (M_diff_=− 6.92, *p* = .03) and the proportion of women was significantly higher (74% vs. 46%, *p* < .05) compared to the control group. No significant differences between groups were observed regarding years of education or educational level. Fourteen participants had adjuvant treatment with radiotherapy. Ten patients received chemotherapy within one-year post-surgery (i.e., temozolomide (TMZ), and one patient received adjuvant procarbazine, lomustine and vincristine (PCV) as well). Sixteen participants in the intervention group (70%) and 20 participants in the control group (77%) chose to involve an informal caregiver. Mean pre-intervention scores of cognitive performance and PROs did not differ significantly between groups (Table 1).

**Table 1:** Sociodemographic and clinical characteristics of intervention group and control group

Characteristic	Intervention group (*n* = 23)	Control group (*n* = 26)	*p* value
Age at T3 (Mean; SD)	45.7 (11.7)	52.6 (10.4)	0.033*
Sex (*n* female; %)	17 (74)	12 (46)	0.048*
Years of education (Mean; SD)	15.4 (3.6)	15.1 (3.6)	0.766
Level of education (*n*; %)			0.334
Low	4 (17)	5 (19)	
Middle	4 (17)	9 (35)	
High	15 (65)	12 (46)	
Physical status (*n*; %)^a^			1.00
ASA I/II	23 (100)	25 (96)	
ASA III/IV	–	1 (4)	
Tumor histology (*n*; %)^b^			0.821
Grade 1 meningioma	13 (57)	14 (54)	
Grade 2 meningioma	1 (4)	1 (4)	
Grade 2 glioma	9 (39)	10 (39)	
Grade 3 glioma	–	1 (4)	
Tumor hemisphere^c^ (*n*; %)			0.681
Left	11 (48)	11 (42)	
Right	11 (48)	14 (54)	
Bilateral	1 (4)	1 (4)	
Tumor localization^d^			0.240
Frontal	13 (57)	11 (42)	
Parietal	3 (13)	2 (8)	
Temporal	5 (22)	7 (27)	
Occipital	2 (9)	1 (4)	
Parieto-occipital		2 (8)	
Temporal-parietal		1 (4)	
Temporal insular		2 (8)	
Radiotherapy after surgery^a,e^ (*n*; %)	4 (17)	10 (39)	0.103
Chemotherapy after surgery^e^ (*n*; %)	3 (13)	7 (27)	0.299
Psychotropic medication at T3^f^ (*n*; %)	11 (48)	17 (65)	0.215
Cognitive impairment^g^ at T3 (*n*; %)	16 (70)	16 (69)	0.980
Low PRO scores^h^ at T3 (*n*; %)	14 (61)	18 (69)	0.539
Involvement of informal caregiver	16 (70)	20 (77)	0.339

*ASA* American Society of Anaesthesiologists,* PROs* patient reported outcomes

^a^Fisher’s Exact Test was interpreted, since not all cell counts were greater than five

^b^Proportions of patients with meningioma and glioma were compared between groups (not separated by tumor grade)

^c^Patients with bilateral tumors were excluded for the statistical comparison

^d^Proportions of patients with tumors with frontal involvement (vs. non-frontal involvement) were compared between groups

^e^During study participation (i.e. within one-year post-surgery)

^f^Use of anti-epileptic drugs, corticosteroid drugs, benzodiazepines, opioids, antipsychotics, stimulants and/or antidepressants

^g^*Z* score ≤ − 1.5 on one or more performance-based outcomes

^h^*Z* score ≤ − 1.5 on one or more PROs

### Adherence and patient satisfaction

Participants completed on average 85% of the strategy training and 91% of the retraining. Sufficient adherence (completion of ≥ 80% of both the strategy training and retraining) was observed in 16 participants (70%). Furthermore, 14 participants completed the retraining more than once.

The evaluation questionnaire was fully completed by 21/23 participants, and partly by one (Table 2). 90% of participants rated to program as “good” or “excellent”, and 95% indicated that they would recommend the program to others. Overall, the level of difficulty and amount of information in the strategy training was perceived as sufficient and participants indicated that the information was useful. However, 14 indicated that there were (too) many fill-in exercises included in the strategy training. Furthermore, 11 reported that there were (too) few exercises included in the retraining and 13 that the retraining was (too) easy. Nevertheless, these exercises were perceived as (very) useful by 19 participants. All participants indicated to have appreciated the tablet-based delivery of the program and 16 indicated that the (telephone) contact was (very) useful.

**Table 2:** Post-intervention ratings of different aspects of *ReMind* (*n* = 22)

Difficulty of	(Too) easy	Just right	(Too) difficult
Information in strategy training	7	13	2
Fill-in exercises in strategy training	9	7	4
Retraining (*C-Car* game)	13	8	–
Amount/number of	(Too) little/few	About right	(Too) much/many
Information in strategy training	1	19	2
Fill-in exercises in strategy training	–	7	14
Retraining exercises (*C-Car* game)	11	10	1
Supervision by the researcher/trainer	–	22	–
Usefulness of	(Very) useful	Neutral	Not useful
Information in strategy training^a^	14	5	2
Fill-in exercises in strategy training^a^	5	10	5
Retraining exercises (*C-Car* game)	19	2	1
(Telephone) contact with the researcher/trainer	16	6	–
Content addressed daily problems	Fully/largely	Partly	Not
	10	7	4
Application of learnt (strategies) in daily life	Often/regularly	Sometimes	Seldom/never
	9	7	6
Impact of cognitive problems has changed	Yes, positively	No^b^	Yes, negatively
	10	11	–
Coping with cognitive problems has changed	Improved coping	No^c^	Worsened coping
	6	16	–
Pleasantness of working on *ReMind*	(Very) pleasant	Neutral	(Very) unpleasant
	7	14	–
	Excellent/good	Sufficient	Insufficient/poor
Using an iPad-app for cognitive rehabilitation	20	2	–
Capability of the researcher/trainer	20	2	–
Contact with the researcher/trainer	21	1	–
Overall rating of the program	19	1	1
	Yes	No	
Recommendation to other brain tumor patients	21	1	

^a^Missing values for two participants

^b^No change, there was no impact on daily life (7) or no change, impact remained the same (4)

^c^Coping is still good (14), or coping is still not good (2)

### Performance-based cognitive outcomes

**Fig. 2:**
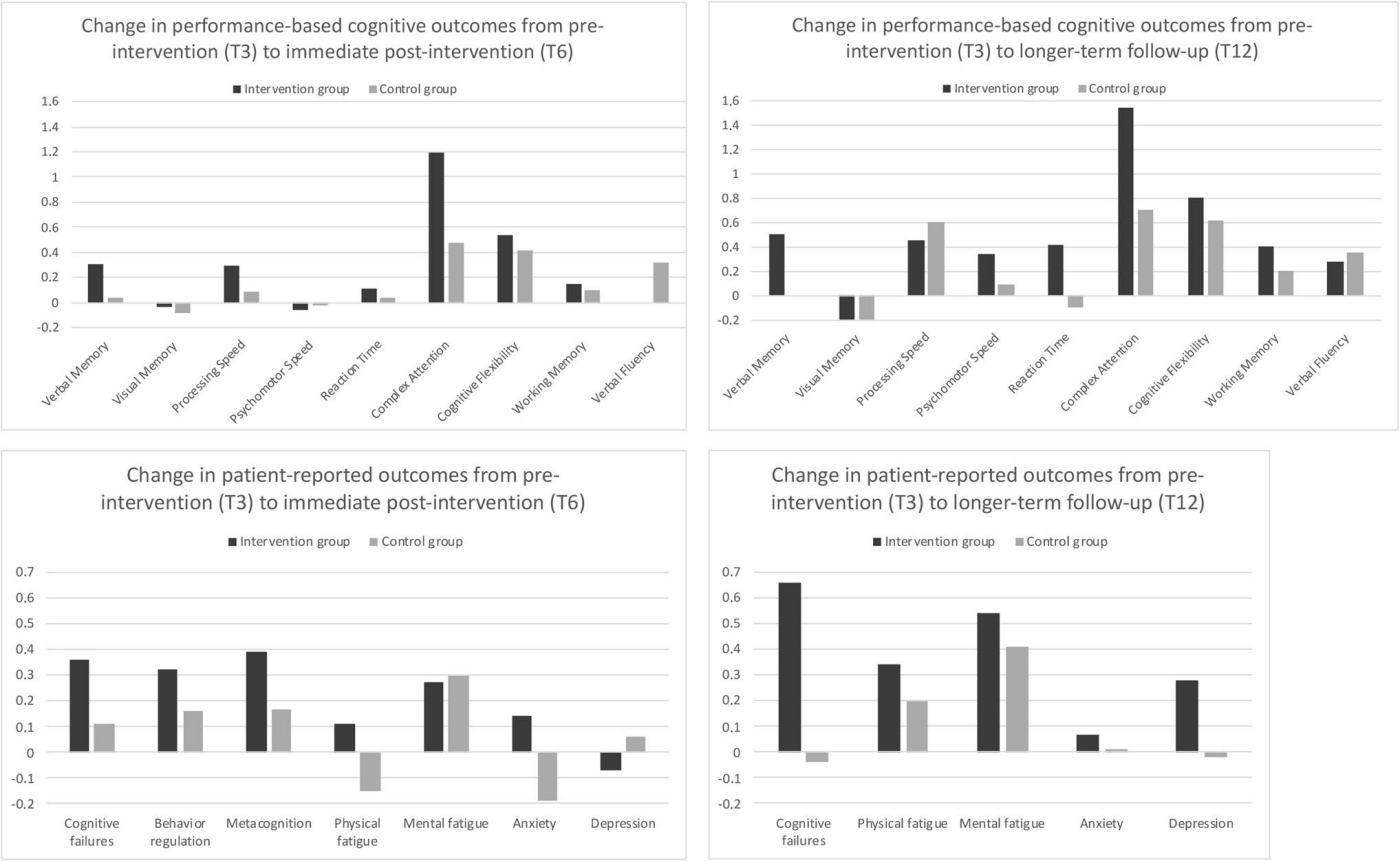
Mean changes in *Z*-scores for performance-based cognitive outcomes and patient-reported outcomes from pre-intervention to immediate follow-up and from pre-intervention to longer-term follow-up for the intervention group versus control group. Positive change scores indicate improvement on the outcome variables, whereas negative scores indicate decline

Mean scores of the groups at the different time points are listed in Table 3 and mean changes over time are presented in Fig. 2. Repeated measures ANOVAs demonstrated no significant interaction effects of time and group on the outcome variables (all *p *values > 0.05). Regarding cognitive performance, significant positive main effects of time (irrespective of group) were observed for processing speed (*F*(2,84) = 8.658, *p* < .001), complex attention (*F*(2,80) = 6.253, *p* = .003), cognitive flexibility (*F*(2,82) = 9.028, *p* = < 0.001) and working memory (*F*(2,78) = 3.147, *p* = .048). Proportions of participants with impairment in cognitive performance were not significantly different between the groups at T3 and T6, with percentages lying around 70% (Table 3). At T12, significantly fewer participants in the intervention group showed cognitive impairment (35% vs. 68%, *p* = .027).

**Table 3:** Mean Z-scores of the intervention group and control group on cognitive performance and PROs per time-point

	Intervention group			Control group		
	T3 (***n*** **= 23)**	T6 (***n*** **= 23)**	T12 (***n*** **= 20)**	T3 (***n*** **= 26)**	T6 (***n*** **= 26)**	T12 (***n*** **= 25)**
Cognitive performance outcomes						
Verbal Memory	− 0.41	− 0.10	0.09	− 0.68	− 0.64	− 0.68
Visual Memory	0.13	0.09	− 0.07	− 0.37	− 0.45	− 0.56
Processing Speed	− 0.36	− 0.07	0.09	− 0.60	− 0.51	0.00
Psychomotor Speed	− 0.22	− 0.28	0.12	− 0.36	− 0.38	− 0.27
Reaction Time	− 0.55	− 0.44	− 0.13	− 1.36	− 1.32	− 1.46
Complex Attention	− 1.54	− 0.35	0.00	− 1.22	− 0.75	− 0.51
Cognitive Flexibility	− 0.98	− 0.45	− 0.18	− 1.19	− 0.77	− 0.57
Working Memory	− 0.06	0.09	0.34	− 0.05	0.05	0.15
Verbal Fluency	− 0.34	− 0.33	− 0.05	− 0.60	− 0.28	− 0.25
Impaired on ≥ 1 performance-based outcomes (*n*; %)	16/23 (70)	15/23 (65)	7/20 (35)	19/26 (73)	18/26 (69)	17/25 (68)
Patient Reported Outcomes						
Cognitive complaints (CFQ)	− 0.43	− 0.07	0.23	0.12	0.23	0.08
Behavioral regulation (BRIEF-A)	− 0.13	0.19	–	0.10	0.26	–
Metacognition (BRIEF-A)	− 0.66	− 0.27	–	− 0.41	− 0.24	–
Physical fatigue (MFI)	− 0.63	− 0.52	− 0.29	− 0.66	− 0.81	− 0.46
Mental fatigue (MFI)	− 0.96	− 0.69	− 0.42	− 1.04	− 0.74	− 0.63
Anxiety symptoms (HADS)	0.12	0.26	0.19	0.18	0.37	0.38
Depressive symptoms (HADS)	− 0.02	− 0.09	0.26	0.19	0.13	0.11
Impaired on ≥ 1 PRO (*n;* %)	14/23 (61)	9/23 (39)	5/20 (25)	18/26 (69)	14/26 (54)	13/25 (52)

Higher mean scores indicate better outcomes

* CFQ* cognitive failures questionnaire, *BRIEF-A * behaviour rating inventory of executive function,* MFI* multidimensional fatigue inventory,* HADS* hospital anxiety and depression scale

Over the first interval (T3–T6), 48 % of the participants in the intervention group and 23% of the participants in the control group showed reliable improvements on one or more cognitive outcomes, and reliable decline was observed in respectively 30% vs. 15%. Between T3 and T12, improvement was observed in 35% of the participants in the intervention group vs. 24% of the controls, and decline in respectively 20% vs. 32% (all *p’s* > 0.05).

### Patient-reported outcomes

For the PROs, no significant interaction effects of time and group on the outcome variables (all *p *values > 0.05) were found. Positive main effects of time were observed, indicating fewer concerns with respect to behavioral regulation (*F*(1,46) = 8.439, *p* = .006), metacognition (*F*(1,46) = 9.149, *p* = .004) and mental fatigue (*F*(2,70) = 4.003, *p* = .022). In the comparison of proportions of impaired scores, no significant between-group differences were demonstrated for any of the time-points.

Over the first interval, 83% of the participants in the intervention group, and 89% of the controls improved reliably on one or more PROs. Reliable decline was observed in 30 and 19% of the participants in the intervention group and control group respectively. Over the follow-up interval, improvements were observed for 85% of the intervention group vs. 72% of the controls, and decline was observed in 10% vs. 20% respectively (all *p’s* > 0.05).

## Discussion

In this RCT, the effects of a tablet-based cognitive rehabilitation program starting three months after neurosurgery were evaluated in 49 adults with low-grade glioma and meningioma. Recruitment of patients before surgery was challenging. Adherence rates were however adequate, with participants completing on average 85% of the strategy training and 91% of the retraining. 90% of participants rated the program as “good” or “excellent”, and 95% indicated that they would recommend the program to other brain tumor patients. In general, means over time for cognitive performance test scores (with corrections for practice-effects) and PROs appeared to improve in both groups, and both improvements and declines were observed at the individual level. However, no significant differences were demonstrated between the intervention group (*n* = 23) and controls (*n* = 26) on group means over time and RCIs.

Our previous RCT, evaluating the face-to-face version of the cognitive rehabilitation program in lower-grade glioma patients with cognitive complaints and disorders, demonstrated positive effects on mental fatigue and performance on memory and attention tests [19]. Several differences in study design may explain findings of the current trial. Compared to our current RCT in 49 brain tumor patients, data from many more patients were evaluated in our previous study (*n* = 135), with much larger statistical power. Initially, we aimed to include 50 patients per group. However, recruitment of participants was difficult, which resulted in underpowered statistical analyses. Unfortunately, recruitment problems are common in psycho-oncological studies, especially in RCTs [43, 44]. Timing of the intervention in the current study may have played a large role here as well. Many patients (52%) were not eligible for inclusion, and additionally, a substantial part of patients declined participation. The majority (56%) mentioned that it would to be too burdensome for them. Of course, the targeted patients face a complex period after surgery. In this period, they need time for recovery and adjuvant treatment, learn to cope with their diagnosis and symptoms, and prefer to devote their time to family, home, work resumption, and social and leisure activities. In our previous RCT, we selected participants based on presence of cognitive complaints and/or deficits and years after treatment [19]. In the current study we chose to adopt an early, preventative and inclusive approach, given that a very large proportion of people with brain tumors experience cognitive deficits at a certain point during the disease trajectory. However, based on our findings, we can conclude that for a substantial group of patients, this early approach does not seem to meet their needs.

Also, not only people with glioma (*n* = 20) were included in the study but also people with meningioma (*n* = 29), who may respond to cognitive rehabilitation in different ways. Unfortunately, the small sample size hampered subgroup analyses on differences in outcomes for meningioma vs. glioma, adherent vs. non-adherent participants and those who involved informal caregivers vs. patients who did not. Furthermore, it is possible that positive preventative effects of the intervention may have been measurable at a later stage (> 6 months post-intervention), which would have required a longer follow-up period.

Furthermore, an eHealth instead of face-to-face cognitive rehabilitation program was used in this study. To our knowledge, this is the first study on eHealth cognitive rehabilitation in brain tumor patients. eHealth has the potential to deliver intervention programs to many patients in a cost-efficient way [45]. Research has demonstrated that psychological eHealth interventions can be as effective as face-to-face programs [46], especially when support is offered, using a blended care approach [47, 48], as in the current trial. A recent practical guideline of the World Health Organization stated that in case of translation of a non-digital validated intervention to a digital intervention, evaluation can focus on the performance of the digital health intervention’s delivery system [49]. In our study, all patients appreciated the tablet-based delivery of the program, all indicated that the amount of supervision (two-weekly phone calls) was ‘about right’, and their adherence to the program was adequate.

In sum, a large number of variables may explain the differences in findings between the previous and the current trial. These include (a) patient factors (differences in brain disease, the stage and duration thereof, presence of cognitive impairments and cognitive complaints, patient needs and motivation, and timing of medical treatments); (b) intervention factors (e.g., preventative versus rehabilitative aim/approach, mode of delivery, intensity of therapist contact, involvement of significant others) as well as (c) methodological variables (differences in sample sizes, possible statistical techniques, and types of outcome assessment). These factors, additionally including sociodemographic factors (e.g., age, education), other intervention (e.g., duration, frequencies and number of sessions) and study factors (e.g., control for practice and other non-treatment effects and other non-treatment effects), may also explain why mixed results have been found in general within the larger field of cognitive rehabilitation research in these and other patient populations [18, 39–42].

A notable finding in our study was that at T12, a significant difference in proportions of individuals with cognitive impairment was found between the intervention group (35%) and controls (68%), while percentages between groups were comparable at T3 and T6 (±70%). This may be partly explained by the observation that pre-intervention scores of the patients in the intervention group seemed already, although not statistically, slightly higher: Post-surgical cognitive improvements over time may have led to a higher percentage of patients in the intervention group reaching normal (non-impaired) ranges at T12. The fact that the groups were not equal for age and sex may have contributed to this. On the other hand, the differences in proportions on T12 may also suggest small beneficial effects of *ReMind*, in combination with the observed non-significant differences in change scores over time in favor of the intervention group.

In the meantime, a feasibility study (*n* = 12) by the University of California, San Francisco, demonstrated that the English version of *ReMind* was well received by adults with lower-grades glioma. Currently, a feasibility clinical trial is ongoing in the same center in which participants with lower grade gliomas are first offered a one-on-one cognitive rehabilitation option (*n* = 20). If they decline due to logistical reasons, they are randomly assigned to *ReMind* (*n* = 20), or to an automated texting program (*n* = 20). Changes in cognition and HRQOL will be correlated with serial imaging at pre-intervention compared to short and intermediate-term follow-up (e.g., in T2 flair hyperintensity volume, diffusion, and resting-state fMRI) [50]. Results of the study are awaited.

To recapitulate, the difficulty recruiting patients for this cognitive rehabilitation intervention suggests that cognitive rehabilitation at this early stage of the disease does not meet the needs of all adults with brain tumors. Patient-tailored timing of *ReMind* (with respect to medical treatment, presence of cognitive impairments and cognitive complaints, and motivation) could have resulted in a larger sample and more reliable statistics, perhaps with more positive findings as the data cautiously suggest. In clinical practice, neuropsychological interventions can be better tailored to the needs and circumstances of patients and family members, and rehabilitation goals can be set in collaboration. Also, more work on treatment options for cognitive deficits in adults with brain tumors is needed, wherein eHealth can be a promising tool.

## Data Availability

Research data will not be shared.
